# Challenges for Upcycled Foods: Definition, Inclusion in the Food Waste Management Hierarchy and Public Acceptability

**DOI:** 10.3390/foods10112874

**Published:** 2021-11-20

**Authors:** Hanieh Moshtaghian, Kim Bolton, Kamran Rousta

**Affiliations:** Swedish Centre for Resource Recovery, University of Borås, 50190 Borås, Sweden; kim.bolton@hb.se (K.B.); kamran.rousta@hb.se (K.R.)

**Keywords:** upcycled food, waste to value food, value-added surplus food, valorised food, food waste management hierarchy

## Abstract

Upcycled foods contain unmarketable ingredients (e.g., damaged food produce, by-products and scraps from food preparation) that otherwise would not be directed for human consumption. Upcycled food is a new food category and thus faces several challenges, such as definition development, inclusion in the food waste management hierarchy and public acceptability. This review provides an overview of these three challenges. The upcycled food definitions have been developed for research, food manufacturers, and multi-stakeholders use. Thus, there is a need for a consumer-friendly definition for the general public. A simplified definition is proposed to introduce these foods as environmentally friendly foods containing safe ingredients that otherwise would not have gone to human consumption such as damaged food produce, by-products and scraps from food preparation. Moreover, an updated version of the food waste management hierarchy has been proposed by including the production of upcycled foods as a separate waste management action that is less preferable than redistribution but more favourable than producing animal feed. Furthermore, consumer sociodemographic characteristics and beliefs, as well as food quality cues and attributes, were identified as crucial factors for the public acceptability of these foods. Future research should address these challenges to facilitate the introduction of upcycled foods.

## 1. Introduction

Food waste is a worldwide concern. One-third of produced food is wasted each year, resulting in food insecurity, financial loss, and negative environmental impacts [1,2,3]. The world’s annual edible food waste is estimated to be 1.3 billion tonnes [1]. Thus, decreasing food waste provides an opportunity to feed people in need and reduce food insecurity [4]. Moreover, since the economic cost of food waste is anticipated to be $750 billion in a year [1], food waste management can result in financial benefits. Furthermore, food waste contributes to global warming by producing 4.4 billion tonnes of CO_2 eq_ per year [5]. Therefore, in addition to food security and financial advantages, appropriate food waste management will have environmental benefits.

Food waste occurs at all stages of the food supply chain, e.g., production, storage, processing, distribution/transport, retail, foodservice and consumption [6]. In Europe, around 40% of food waste occurs at the production, handling/storage, and processing stages and 60% occurs at the distribution, retail and consumption stages [7]. One of the strategies to manage food supply chain waste is to repurpose the edible part of the wasted food to produce food for human consumption. Therefore, the wasted food will, in fact, not be wasted but will act as a resource for food production. One example of this practice is the production of biscuits from sunflower flour [8] or apple pomace [9]. Foods made from such ingredients are value-added products and are classified as “upcycled foods” [10], a term that has been recently introduced. Upcycled foods are made from unmarketable ingredients such as sub-grade, damaged or imperfect food produce, food by-products and scraps from food preparation [11]. The production of upcycled foods is beneficial to the environment as it helps to repurpose food, that would otherwise be wasted, as a value-added food product [10]. Value-added foods are foods that are produced, processed or altered in a manner that increases their economic value [12,13].

Upcycled food is considered a new food category alongside conventional and organic foods [14]. Since upcycled food is a new concept, it faces several challenges, such as definition development, inclusion in the food waste management hierarchy and public acceptability. The researchers and other stakeholders developed several upcycled food definitions for different purposes, i.e., research, food manufacturers and multi-stakeholders use [10,11,14,15,16]. A coherent and clear definition is critical for communicating climate change-related concepts to the general public [17]. Furthermore, upcycled food production should be incorporated in the food waste management hierarchy to acknowledge its value as a viable food waste management approach. The food waste management hierarchy plays an important role in establishing policies [18]; therefore, the inclusion of upcycled food production in this hierarchy helps to develop policies and regulations. The regulation of upcycled food is also expected to improve its public acceptability. Achieving public acceptability is a challenge for upcycled foods [19] as the main aim of manufacturing these foods is the consumption by the general public.

To our knowledge, there are a limited number of literature reviews on the non-biotechnological aspect of upcycled foods [20,21], and none of them addresses all of the challenges mentioned above. Therefore, this review provides an overview of upcycled food definitions, food waste management hierarchies (for positioning upcycled food production in these hierarchies) and upcycled food acceptability factors.

## 2. Defining Upcycled Food

Converting the edible parts of wasted food into upcycled foods is not a new practice. Taking leftovers and converting them to foods, and recycling ingredients from surplus foods to produce another type of food are some examples [22,23]. Although no specific term for this practice previously existed, its description is similar to upcycling. In upcycling, a new value-added or high-quality, sustainable product will be produced by converting waste/used materials or by reusing a product in a new way while minimizing unnecessary resource expenditure [24]. In the absence of official terminology and a definition for the foods produced by this approach, the term “upcycled food” was recently introduced. The upcycled food definition varies depending on the purpose. It has been defined for conducting research [14], and for food manufacturer use [10] and multi-stakeholder (e.g., experts in the fields of food loss and waste, marketing, law and regulation, government, and the non-profit sector) use, including third-party certification [11]. These definitions describe the sources of upcycled food ingredients from different perspectives. Table 1 provides a summary of these upcycled food definitions.

Some research studies refer to upcycled foods as waste-to-value food products [25] or value-added surplus products [14]. For example, in a study conducted by Bhatt et al. [14] upcycled foods were initially referred to as value-added surplus products and later labelled as upcycled foods due to consumer preference. According to this market research-driven definition, food surplus and ingredients obtained from food manufacturing, including by-products can be used for upcycled food production [14]. Surplus food can be generated at any stage of the food supply chain because it comprises edible food that has been produced, manufactured, retailed or served but has not been consumed [26]. The safety of upcycled foods depends on the source of the ingredients, and thus the stage of the food supply chain in which ingredients are obtained is important. In terms of by-products, industry waste is considered a by-product if its further use is certain [27]. In other words, the intended destination of by-product is not disposal, and thus it is not a waste but rather a resource. This is an important distinction to establish between waste and a by-product, since the consumer acceptability of upcycled food may be affected if the ingredients are viewed as resources rather than waste. Some studies [15,16] have referred to by-products as a source of ingredients for upcycled foods to facilitate consumer understanding of this new food category.

From a manufacturer’s point of view, upcycled food production is an approach for upgrading food that would otherwise be wasted to food for human consumption [10]. This manufacturer-driven definition emphasizes upcycled food production as a superior approach to non-food usage and focuses on the environmental benefits of this practice as an advantage. An “elevation to a higher use” refers to upgrading the ingredient’s use from animal feed, compost and disposal to human food [10]. Using the term “food that would otherwise be wasted” can positively impact the public acceptability of upcycled foods because it refers to wasted food rather than food waste. Wasted food implies the loss of food as a valuable resource, whereas food waste conveys the generation of valueless materials as waste [28,29]. It is worth mentioning that manufacturers expressed concerns with regard to the practical aspects of the definition, including uncertainties over the acceptable quantity of upcycled ingredients in a food that would allow it to be classified as an upcycled product, and the need for the third-party certification [10].

The multi-stakeholder definition, developed by the Upcycled Food Association, considers specific features of upcycled foods [11]. Such foods should be value-added products for human consumption, should source their ingredients from materials that would otherwise be allocated for non-food destinations, have an auditable supply chain, and declare the upcycled ingredients on their labels [11]. The Upcycled Food Definition Task Force developed this definition for use by industry stakeholders, the governmental sector, policymakers, and for academia [11]. Furthermore, the application of the term “upcycled food” has been recommended for food labelling [11]. The Upcycled Food Association definition appears to be a comprehensive version that includes the essential aspects of the other two definitions. This definition elaborates on the source and alternative destination of upcycled food ingredients. As mentioned, the ingredients are materials that would not otherwise be allocated for human consumption, regardless of whether their originally intended destination was disposal, or the production of other substances. Therefore, the term “waste” has been removed from the definition, and the provision of an auditable supply chain has been added as a criterion to address the food safety concern.

The literature generally refers to food surplus, loss and waste as food waste unless it is necessary to differentiate between these terms [30]. The definition of food waste is subject to debate, and inconsistencies exist with regard to what should and should not be considered as food waste [3,28,31,32,33,34,35]. For example, one definition refers to food waste as “food such as plate waste (i.e., food that has been served but not eaten), spoiled food, or peels and rinds considered inedible that is sent to feed animals, to be composted or anaerobically digested, or to be landfilled or combusted with energy recovery” [28]. Another definition excludes food diverted for animal feed and defines food waste as “any food and inedible parts of food which is removed from the food supply chain to be recovered or disposed (including composted, crops ploughed in/not harvested, anaerobic digestion, bioenergy production, co-generation, incineration, disposal to sewer, landfill or discarded to sea)” [35]. Although this definition does not consider food that is upcycled or diverted to animal feed as food waste [35], upcycling or the diversion of food waste for use in animal feed is one of the main food waste management strategies [30,36,37,38,39,40,41,42,43]. Defining upcycled ingredients as food that otherwise would not have been used for human consumption means that these ingredients can originate from food surplus, waste or loss.

The Upcycled Food Association has recently introduced a program to certify upcycled ingredients and foods, and to make it more feasible for manufacturers to produce these foods [44]. According to this program, to be certified, upcycled ingredients should consist of at least 95% (by weight) uniformed diverted materials [44]. The certified upcycled ingredients cannot be directly sold to consumers but can be used as a food ingredient [44]. Certified upcycled food products should contain at least 10% upcycled ingredients by weight and can be sold to consumers or used as an ingredient for other food products [44]. If the product does not meet this threshold (i.e., if it contains less than 10% upcycled ingredients), it will be certified as a product with minimal upcycled ingredient content [44]. This program also considers the food safety and environmental impact assessment of the upcycled foods and ingredients [44]. According to certification requirements, the manufacturer should provide information on the production process, its traceability, a risk assessment, and greenhouse gas emission [44].

The current definitions describe upcycled foods from the perspectives of researchers [10,14,15,16] and other stakeholders [10,11]. Although the Upcycled Food Association [11] definition appears more comprehensive than the others, the general public may have difficulty understanding this definition. In general, people in the community may not clearly understand climate change-related terminologies [17]. Thus, a definition that contains simple words and avoids complex language can be more effective for communication and comprehension by the general public [17]. Therefore, the upcycled food definition needs to be further simplified to aid consumer understanding, education and the acceptability of these products. For example, upcycled foods can be defined as *environmentally friendly foods containing safe ingredients that otherwise would not have gone to human consumption such as damaged food produce, by-products and scraps from food preparation*. Future research should assess the consumers’ understanding of this proposed definition and revise it if necessary. A simple definition accompanied by certification that confirms the positive environmental impact and safety of upcycled food is expected to enhance public acceptability.

## 3. Upcycled Food Production in the Food Waste Management Hierarchy

### 3.1. Food Waste Management Hierarchy

The concept of a waste management hierarchy was introduced to provide guidance for the order of waste management actions [45]. This concept was developed to prioritize waste reduction, reuse and recycling over treatment and disposal [46]. The general waste management hierarchy has five stages, from the most to least preferred actions, and includes prevention, reuse, recycling, recovery and disposal [27,47]. The EU Waste Framework Directive encourages actions with the best environmental impacts, and recommends making a selection by considering resource and environmental protection as well as health, social and economic impacts [27]. The priority order of actions in the waste management hierarchy is based on “assumed” environmental impacts, and the results from lifecycle assessment (LCA) studies of the actual environmental impact have been inconclusive [46]. LCA often considers a simple and generalised scenario and has uncertainties in mapping the resource use, emissions and impact [48]. The LCA calculated impact is also affected by time and location/country [48]. Therefore, it is difficult to have a single LCA-based waste management hierarchy that is relevant in all parts of the globe, for all types of waste. The waste management hierarchy is also applicable to food waste management. However, food waste has specific characteristics and potentials, and thus a general and simple hierarchy cannot cover all the possible management strategies [49]. As a result, various versions of the food waste management hierarchy have been introduced in the literature.

Based on the aim and perspective of researchers and stakeholders, hierarchies have been specifically tailored and revised to address various food waste management strategies. A summary of these hierarchies and their perspectives are presented in Table 2. Teigiserova and Hamelin [30] and the European Commission’s Knowledge Centre for Bioeconomy [40] focused on the economic gains of the hierarchy, whereas, US Environmental Protection Agency [39], United Nations Environment Programme [41], UK Department for Environment, Food and Rural Affairs [42], Dutch Ministry of Agriculture, Nature and Food Quality [43], and Zero Waste Europe [49] presented hierarchies to guide stakeholders and the public regarding the priority order of food waste management actions. The food waste management model introduced by Garcia-Garcia, et al. [37] supports the waste management decision-making process by elaborating on the different stages of the hierarchy, specifically the end-of-life stages. The hierarchy developed by Papargyropoulou et al. [36] focuses on differentiating between food surplus and food waste management. The food surplus is suitable for human consumption, and when it becomes inedible, it turns into waste [36].

Different food waste management perspectives create inconsistencies in the number and order of the waste management actions within the hierarchy. Some hierarchies [30,38,39] provide a general model with a small selection of actions, resulting in different interpretations of each action. Other hierarchies are detailed and describe a multitude of scenarios [37,43]. Although the first three steps of the hierarchies listed in Table 2 are similar, some contradictions exist in the order of actions that occur lower in the hierarchy. For example, in some models, composting is preferred to anaerobic digestion [36,38] and vice versa [37,41]. In another model, both actions are placed at the same level in the hierarchy [49]. This might be due to the intended use of the hierarchy, because composting is applicable at both industrial and household levels, whereas anaerobic digestion appears to have more environmental benefits [50]. Furthermore, incineration with and without energy recovery has been included in some of these hierarchies [37,39,40,41,49]. The dried food waste can be incinerated in mixed solid waste, resulting in energy recovery [51]. However, incineration without energy recovery and landfilling are not recommended as disposal actions [50].

### 3.2. Inclusion of Upcycled Food Production in Food Waste Management Hierarchy

Upcycled food production has been recently introduced as a food waste management option, and thus it is essential to consider upcycled food in the food waste management hierarchy. From the animal feed stage of the hierarchy onwards, the wasted food is no longer intended for human use [40,52]. Since upcycled foods are intended for human consumption, the upcycled food production should precede animal feed. This section reviews food waste management actions before the animal feed stage to appropriately position the upcycled food production in the hierarchy.

The most preferred action in the food waste management hierarchy is prevention, which refers to avoiding food waste generation throughout all stages of the food supply chain, and food surplus generation at the production and consumption stage [36]. Upcycled food production is not a food waste prevention action because producing these foods is a strategy for utilising the generated food waste/surplus. In other words, if the prevention is 100% (no waste), there will not be any material to be used as an ingredient for upcycled food production. With regard to the hierarchy of actions, food waste prevention is preferable to upcycled food production because food processing requires energy and natural resources (e.g., water) and thus has adverse environmental impacts [53]. Hence, the production of upcycled food should appear below prevention in the food waste management hierarchy. It is worth mentioning that off-grade or surplus foods (e.g., fruit and vegetables) cannot be deemed as upcycled foods when they are only retrieved from a field or diverted from a landfill [11]. These foods can be labelled as upcycled if they are processed and transformed into a new product [11].

The second preferred action in the food waste management hierarchy is food redistribution. Food redistribution occurs when surplus food is donated to charity organisations, redistribution networks and food banks [37,54]. This is usually defined as the donation of food from farmers’ fields, retail stores and foodservice facilities [55]. The hierarchical models in Table 2 used different terms such as reuse [30,36,40], food recovery [49] and redistribution [37,41] for the second stage; however, the description of these terms refers to donation. It has been suggested that value-added food production could be considered within the human reuse stage [52], however, producing upcycled food differs from redistribution. In redistribution, food products are donated without further processing, whereas in upcycled food production, further processing is required to produce value-added foods and to ensure financial gain. In other words, redistribution can be considered reusing food, whereas the production of upcycled foods concerns upcycling the wasted food. As previously mentioned, the production process for upcycled foods can negatively impact the environment and thus should appear below redistribution in the waste hierarchy.

**Figure 1:**
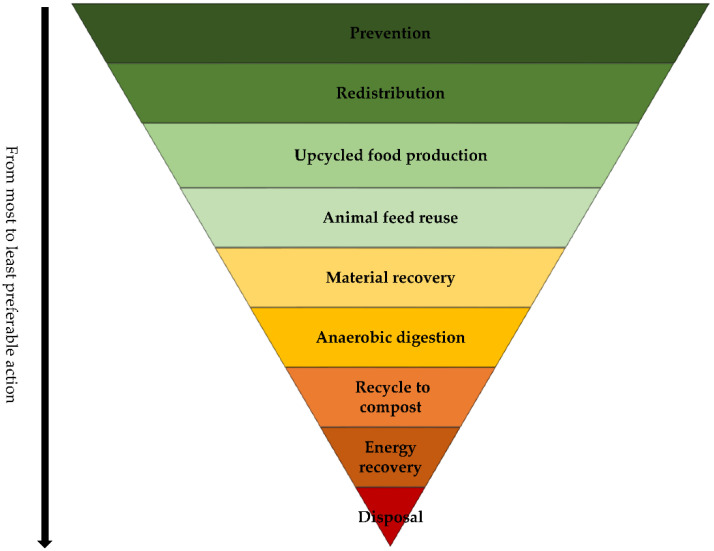
Food waste management hierarchy. The hierarchy for the management of food surplus, waste, and loss [30,36,37,38,39,40,41,42,43,49] has been modified to include upcycled food production as a management action.

Among all food waste management hierarchies [30,36,37,38,39,40,41,42,43,49], only one version of the hierarchy has considered the conversion of wasted food into human food as a separate stage [43]. In this stage, the food is produced by processing or reprocessing wasted ingredients [43]. From a review of these hierarchies, it can be concluded that this stage should be added to the current food waste management hierarchies to address the upcycled food production. The production of these foods should be considered as the third stage of the hierarchy (after redistribution), conveying that redistribution/donation is preferable to upcycled food production. It also means upcycled food production is preferred to animal feed production. This ordering is logical since, by producing upcycled foods, the wasted food will be returned to the food supply chain as human food and will not be diverted for use in animal feed. The food waste management hierarchy shown in Figure 1 has been revised to include upcycled food production as a management action.

## 4. Public Acceptability of Upcycled Food

Although the definition of upcycled food and its inclusion in the food waste management hierarchy are important challenges, public attitudes towards this food category present the most difficult challenge to resolve. This section discusses the public acceptability of upcycled foods and classifies acceptability factors into consumer sociodemographic characteristics and beliefs as well as food quality cues and attributes. It is worth mentioning that upcycled foods were only produced in a few of the reviewed studies [8,9,56,57,58], and most public acceptability studies were based on hypothetical upcycled foods where people expressed their opinion based on the product descriptions and pictures.

### 4.1. Consumer Sociodemographic Characteristics and Beliefs

Consumers sociodemographic characteristics (e.g., gender, age, income and education), and beliefs such as attitudes towards food waste management and sustainability, and their concern for the environment can affect the acceptability of upcycled foods [22,25,59,60,61]. For example, women are more health-conscious and risk-averse than men [62,63,64] and thus may not be in favour of upcycled foods [22,25,59] as they probably perceive these foods as unhealthy options. Furthermore, education level, income, and age influence the acceptability of upcycled foods [59,60,61,65]. Consumers with a high level of education [61] and a high income level [65] are inclined to choose upcycled foods. However, the purchasing intention of these foods does not seem to increase with age [60,61]. Older people are less inclined towards upcycled foods [60,61], but the comparison of different generations shows that middle-aged consumers, overall, have a lower acceptability of upcycled foods compared to younger and older generations [16]. It is worth mentioning that findings for sociodemographic factors are not consistent in all studies [22,60,66] and thus should be interpreted with caution, as the context of each study and the type of upcycled food under investigation were different.

Public beliefs and attitudes can also influence the acceptability of upcycled foods. According to the theory of planned behaviour, people’s behaviour is linked to their beliefs and attitudes [67]. For example, consumers’ preferences for environmentally sustainable foods, such as organic foods, can motivate them to purchase upcycled foods [60]. Additionally, individuals who are concerned about the consequences of food waste [65] and those who are aware of their own food waste generation [22] are also motivated to choose this type of food. These groups may be more sensitive to food waste issues [22] and thus consider upcycled food production as a food waste management option. Furthermore, other factors such as consumers’ food processing experience, risk and benefit perception, trust in manufacturers and regulatory authorities, and food safety, sustainability and ethical considerations were identified as valorised food acceptability factors [22]. The valorised foods in this study contained gleaning-based ingredients, food surplus vitamin extracts, and meat products obtained from animals that were fed with eco-feed or by-products [22]. The mentioned factors that contribute to the acceptability of these foods may also be applicable to upcycled foods.

Since upcycled foods contain ingredients that would not otherwise be used for human consumption, food neophobia and technophobia can influence their acceptability. There is a general aversion and neophobia towards novel foods and foods produced by innovative methods [68], and technophobia has been identified as a barrier to consumer acceptance [69]. According to Coderoni and Perito [60], upcycled food purchasing intentions can be affected by consumer food and food technology neophobia, trust and concerns regarding product safety, healthiness and nutritional value. Therefore, food production methods affect the acceptability of these foods, and unfamiliarity with the production process contributes to reluctance towards them [22]. Likewise, unfamiliarity with upcycled ingredients can affect food acceptability. For example, the consumer’s low acceptability of protein drinks made from potato by-products or grass as a protein source [25] can be due to the inclusion of unfamiliar ingredients.

### 4.2. Upcycled Food Quality Cues and Attributes

Food quality affects consumer acceptability [70] and is determined by cues and attributes [71]. The food quality cues are based on intrinsic (e.g., colour, appearance, structure, shape and size) and extrinsic (e.g., brand, nutrition and production information /labels, presentation, store, price and country of origin) cues [71,72,73]. Food quality attributes are related to experience quality factors such as taste, freshness and convenience, and credence quality factors e.g., healthfulness, nutritional value, naturalness, production methods, and animal and environmental friendliness [71,72,73]. Food marketing messages focus on these cues and attributes to attract consumers.

#### 4.2.1. Food Quality Cues

Food intrinsic cues are mostly ingredient-related and thus cannot be easily altered without modifying the physical characteristics of the product [14,71]. Intrinsic cues are used in food sensory evaluation [74], and some studies have developed an upcycled food product to assess its intrinsic cues such as appearance and colour [8,56,57,58]. These studies demonstrated that incorporating upcycled ingredients into the food product formulation influences its intrinsic cues [8,56,57,58]. For example, upcycled biscuit appears darker or less aerated than conventional alternatives [8]. Therefore, it may be challenging to improve some of the upcycled food intrinsic cues.

Since upcycled food intrinsic cues are difficult to modify, marketing activities mainly focus on extrinsic cues [14] such as labels, certification, country of origin and price [60,61,75]. Consumer attention to the food labels, certification, list of ingredients and country of origin affects upcycled food acceptance [60,75]. The certification of upcycled foods with carbon footprint labels such as carbon trust improves their acceptability, particularly among environmentalists [19]. The recent certification of upcycled foods by the Upcycled Food Association [44] may also improve the acceptability of these foods as a well-designed logo acts as a food quality indicator [76]. In terms of product brand, although consumers consider brand as one of the food quality indicators [77], this factor does not appear to act in favour of upcycled foods [25,60].

Another extrinsic cue is price, which plays a dual role in consumer choices [78,79]. A high price may decrease the probability of purchasing, but conversely, indicates high quality and thus positively impacts the perceived utility and demand [78,79]. The high price of environmentally friendly foods [80] affects their affordability for the public, particularly for low-income consumers [81]. The willingness to pay for upcycled foods depends on the public perception of these foods and on marketing communication. For example, if the upcycled food is perceived to have the same quality as a conventional product, people will be willing to pay a premium price [61]. Since upcycled food production is a food waste management approach, consumers accept the higher price for this effort [61]. Similarly, consumers are willing to pay a premium price when the upcycled food is introduced as a food with nutritional and environmental benefits [82]. However, when the marketing message focuses on the ingredient source (e.g., food waste and sub-optimal ingredients), people expect to pay a discounted price for such foods [15]. Therefore, transparency in upcycled food communication does not necessarily convince consumers to buy or pay more, and, in fact, it may negatively affect their choices [83].

#### 4.2.2. Food Quality Attributes

Food quality attributes (i.e., experience and credence quality factors) [71] can also influence the public acceptability of upcycled foods. Investigating the acceptability of hypothetical upcycled foods provides valuable insight; however, the impact of hedonic characteristics should not be ignored. Taste is one of the major predictors of consumer attitudes towards a food [84]. Some upcycled food studies evaluate sensory aspects, particularly taste and texture, for products such as cereal bars made from brewery spent grains [58], fungi burgers from surplus bread [56] and biscuits from defatted sunflower seed [8], apple pomace [9], and pineapple, apple and melon by-products [57]. These studies revealed that the sensory characteristics of upcycled foods are conditional and depend on the product formulation and the proportion of upcycled ingredients [8,9,56,57]. The taste and texture of an upcycled food may differ from conventional products, and thus these foods appear more [57] or less favourable [8,56,58] than their conventional alternatives. These sensory features affect consumer willingness to incorporate upcycled foods into their daily diet and to replace conventional foods. In studies regarding hypothetical upcycled foods, consumers did not taste the food and thus, the perceived acceptability and purchasing intention is conditional.

As mentioned previously, upcycled foods contain food waste/surplus derived ingredients, and therefore some of their features may be difficult to improve. However, the presence of quality attributes such as nutritional value, healthfulness, and environmental benefits can increase the acceptability of these foods [82,85]. Some upcycled ingredients are high in protein [8], fibre [9,57] and antioxidants [8] and have a low glycaemic index [9]. Therefore, they can have a positive impact on human health. For example, proteins are protective against sarcopenia (loss of muscle mass) and frailty [86]. Dietary fibre is associated with cardiovascular and gastrointestinal health [87]. Antioxidants prevent cancers, neurodegenerative disease (e.g., Alzheimer’s and Parkinson), diabetes, hypertension and cardiovascular disease [88]. Low glycemic index foods decrease the risk of developing diabetes and cardiovascular disease [89].

The success of nutritional messages depends on the food nutrient content. Hence, these messages may not be applicable to all upcycled foods. Nutrient fortification is a strategy used to improve food quality [90], and upcycled foods can take advantage of this approach to improve their acceptability. Consumers prefer upcycled foods over conventional products when communication emphasizes high protein content (nutritional benefit) and a low carbon footprint (environmental benefit) [82]. Communication of the product’s environmental benefits can influence public attitude towards these foods because consumers believe upcycled foods are more environmentally friendly than conventional alternatives [14]. Both nutritional and environmental benefit messages appear to be influential, and environmental messages may not be more effective than nutritional messages [82].

## 5. Critical Discussion

This review provided an overview of various upcycled food definitions from research and stakeholder perspectives and suggested a simplified definition for the general public. Moreover, it discussed the food waste management hierarchies and proposed the inclusion of upcycled food production in the hierarchy. It also reviewed consumer-related factors and food quality characteristics that influence upcycled food acceptability. Upcycled food production is a viable approach for food waste management, thus, the introduction of this food category to the general public should be facilitated. An appropriate introduction of upcycled foods to stakeholders and consumers, accompanied by policy and regulation development can improve their acceptability. Upcycled foods are value-added products, and therefore, if their production also results in the development of healthy, nutritious, and environmentally friendly foods, people may be more inclined to purchase and consume these foods.

A consumer-friendly definition of upcycled food can facilitate the introduction of these foods to the general public. The current definitions consider different aspects of upcycled foods such as food waste management, environmental benefits and safety [10,11,14]. However, some of these aspects should be confirmed in relation to each upcycled food product. For example, the positive environmental impact of upcycled foods depends on the results of LCA studies. Two points should be taken into account when assessing the positive environmental impact of upcycled foods in LCA studies. The first point is whether upcycled food production has a more positive environmental impact compared to conventional food production. The second point is whether the diversion of food surplus, waste and loss, for upcycled food production, has a positive environmental impact compared to their diversion to other food waste management destinations (e.g., animal feed production). Some environmental issues related to food manufacturing include waste generation, excessive use of resources (energy and water) and a high carbon footprint [53,91]. The upcycled food certification program does not require the manufacturer to provide a full LCA, but it asks for calculating greenhouse gas emissions to evaluate the environmental impact of upcycled food production [44].

In terms of food safety, including a verifiable and auditable supply chain in the upcycled food definition can help consumers accept these foods. Although the ingredient sources of these foods are food surplus, by-products and waste from food preparation [11], they must comply with food safety legislation. All food products should meet specific criteria to be eligible for market release [92,93]. For example, they should comply with food safety protocols such as the Hazard Analysis Critical Control Point (HACCP) [94]. The same rules apply to upcycled foods, and thus their market release implies their compliance and safety. If these foods do not meet the food safety criteria [92,93] or novel food regulations [95], they will not reach the consumers. In this case, they may be viable for use as animal feed. However, the eligibility for conversion to animal feed depends on whether materials meet the animal feed safety regulations [92].

One of the advantages of upcycled food production is its contribution to food security [16]. The four pillars of food security are availability (availability of food supply), access (physical and economic access to food), utilization (utilization of food), and stability (stability of food supply and access) [96]. The production of upcycled foods positively impacts food availability as it addresses the supply pillar of food security. If the manufacturers offer discounted prices for these products, the affordability, and thus the accessibility aspect of food security, will also be addressed. Some people believe that upcycled foods should be discounted as they contain suboptimal ingredients [22,65]. However, since they are value-added products [10], they may not be cheaper than conventional alternatives. Furthermore, if upcycled food production reduces redistribution/donation, it will be disadvantageous to people in need and negatively affect food security. Regarding the utilization pillar, using an auditable supply chain and ensuring food safety can address the consumer nutritional needs and thus food utilization. Food manipulation, additive and seasoning use, and nutrient loss (ultra-processing) during food processing [97] all have a negative impact on the utilization aspect of food security.

Although some consumers believe that upcycled foods are more beneficial than conventional alternatives [14], the healthfulness of these foods has not been thoroughly investigated. Value-added foods contain ingredients that have been processed to increase their economic value [12,13]. This practice does not necessarily increase upcycled food nutritional value and thus does not mean they are healthier than conventional alternatives. If upcycled foods are fortified, or the upcycled ingredients are more nutritious than conventional alternatives, they can serve as healthier options compared to conventional choices. It is worth mentioning that some processed foods, in particular highly processed foods, are high in fat, sugar and salt and are thus considered unhealthy [98]. Saving the environment should not be at the expense of consumers’ health. The production of upcycled foods using healthy and minimally processed ingredients combines sustainability and healthfulness, and thus facilitates consumers’ decision making.

The public knowledge of upcycled foods is limited [19], therefore, communication and education can improve the acceptability of these foods [25]. Communication can address the negative perception towards these foods and emphasize their potential benefits, such as naturalness and environmental friendliness, and thus facilitate consumer acceptance [22]. Furthermore, improving the transparency of the production process, addressing consumers’ moral considerations, and conveying health and nutrition messages increases public acceptance and willingness to purchase these foods [22,82]. Although food industry marketing strategies use communication to influence public perception of upcycled foods [82], more time is needed to change people’s attitude towards these foods and thus continuous communication is required to achieve consumer trust and acceptance [22,59].

In this review, most of the upcycled food acceptability studies were based on hypothetical products and focused on selected factors to assess purchasing intention. Consequently, the generalisability of their findings is limited. Since upcycled foods have lower consumer acceptability compared to conventional products [19], future research should identify all important factors (e.g., health-related factors) that influence the public acceptability of these foods. The intended use of upcycled food is human consumption, and the evaluation of consumer perception should therefore be included in the product development process. In other words, prior to the mass production of innovative upcycled foods, consumer attitudes towards these foods should be assessed. Investigating consumers’ criteria for selecting upcycled foods will help the food industry and researchers to develop food products with high acceptability.

## 6. Conclusions

The current definitions of upcycled food were developed for research and stakeholder use, and thus the understanding of these definitions by the general public has not been taken into account. A simplified and consumer-friendly definition should be considered for communication to the general public. For example, upcycled foods can be defined as environmentally friendly foods containing safe ingredients that otherwise would not have gone to human consumption, such as, damaged food produce, by-products and scraps from food preparation. In terms of the food waste management hierarchy, upcycled food production can be included in the hierarchy to present this action as a viable approach for food waste management. Upcycled food should appear below the redistribution and above the animal feed stage of the hierarchy. Regarding the public acceptability of upcycled foods, this review demonstrated that consumer sociodemographic characteristics and beliefs as well as food quality cues and attributes, play important roles. Thus, communicating the positive aspects of these foods may improve public attitudes towards them. Since upcycled food is a new food category, there is significant research potential to address the concerns and challenges in this context and improve the public acceptability of this food category.

## Figures and Tables

**Table 1:** Upcycled food definitions.

Source	Definition	Application	Aim
Bhatt et al. [14]	Foods made from surplus ingredients or ingredients obtained during the manufacturing of other foods that would have been otherwise wasted.	Research	Assessing the effects of product descriptions, labels, and benefits on consumer evaluation of upcycled foods
Spratt et al. [10]	“Upcycled ingredients and food products elevate food that would otherwise be wasted to higher uses, and have tangible benefits to the environment and society.”	Manufacturer	Developing a definition for upcycled food products from a manufacturer perspective
Upcycled Food Association [11]	“Upcycled foods use ingredients that otherwise would not have gone to human consumption, are procured and produced using verifiable supply chains, and have a positive impact on the environment.”	Multi-stakeholder including the third-party certification	Providing industry, academia, government, and other interested parties with a standardised and workable definition

**Table 2:** Food waste management hierarchies from different perspectives.

Source	Waste Management Hierarchy (from Most to Least Favourable Action)	Perspective
Teigiserova and Hamelin [30]	Prevention, human reuse, animal reuse, material recycling, nutrient recovery, energy recovery and disposal (landfill/incineration)	Circular economy
Papargyropoulou et al. [36]	Prevention, reuse (human consumption), recycle (animal feed and composting), energy recovery (via anaerobic digestion) and disposal (landfill)	Management of food surplus and food waste
Garcia-Garcia, et al. [37]	Prevention of food waste generation, redistribution for human consumption, animal feed, extraction of compounds of interest, industrial use, anaerobic digestion, composting, thermal treatment with energy recovery, land spreading, thermal treatment without energy recovery and landfilling	Supporting decision-making process and industrial food waste management
Zu Ermgassen et al. [38]	Reduce food waste, reuse and redistribute, recycle (animal feed and compost), recovery (anaerobic digestion), disposal	Animal Feed
Zero Waste Europe [49]	Source prevention, food redistribution, repurposing, anaerobic digestion and composting, mechanical biological mixed waste treatment and landfill/incineration	Food waste policies
United States Environmental Protection Agency [39]	Source reduction, feed hungry people, feed animals, industrial use, composting, landfill/incineration	Prioritize organization actions to prevent and divert wasted food
UK Department for Environment, Food and Rural Affairs [42]	Prevent, redistribute, make animal feed, recycle (anaerobic digestion), recycle (composting), recycle (land spreading), incinerate to generate energy, incinerate without generating energy, send to landfill or sewer	Statutory guidance for food producers, manufacturers and retailers to prevent food surplus, recycle and dispose of food waste
Dutch Ministry of Agriculture, Nature and Food Quality [43]	Prevention, use for human food, conversion to human food, use in animal feed, raw materials for industry (biobased economy), processing to make fertilizer for co-fermentation (and energy generation), processing to make fertilizer by composting, use for sustainable energy (objective is energy generation), burning as waste (aim is destruction, with possible energy production), dumping	Guideline for optimum utilisation of residual flows based on ethical norms
European Commission’s Knowledge Centre for Bioeconomy [40]	Prevention, reuse for human consumption, reuse for animal consumption, reuse by-products and recycle food waste, recycle for nutrients recovery, energy recovery, disposal (landfill/incineration/sewage)	Food waste valorisation
United Nations Environment Programme [41]	Prevention, optimisation (redistribution to people and animal feed), recycling (anaerobic digestion and compost), energy recovery, disposal (landfill/incineration/sewage)	Guidance for governments, businesses and other organisations to develop food waste management strategies
